# Documented Obstructive Sleep Apnea, Cardiometabolic Phenotype, and Rhythm‐Related Outcomes After Atrial Fibrillation Ablation: An Observational Cohort Study

**DOI:** 10.1002/joa3.70410

**Published:** 2026-06-30

**Authors:** Yazan Mohsen, Iryna Novikov, Shreeram Sabareesan, Kensuke Sakata, Moritz Knitter, Henning Horlitz, Lucas Steffens, Marc Horlitz, Ibrahim Antoun, Florian Stöckigt

**Affiliations:** ^1^ Alliance for Cardiovascular Diagnostic and Treatment Innovation Johns Hopkins University Baltimore MD USA; ^2^ Department of Cardiology, Electrophysiology and Rhythmology Krankenhaus Porz Am Rhein Cologne Germany; ^3^ Department of Cardiology, Faculty of Health School of Medicine, University Witten/Herdecke Witten Germany; ^4^ Department of Cardiology University Hospitals of Leicester NHS Trust, Glenfield Hospital Leicester UK; ^5^ Division of Cardiovascular Sciences College of Life Sciences, University of Leicester, Glenfield Hospital Leicester UK; ^6^ Department of Internal Medicine II University Hospital Bonn Bonn Germany

**Keywords:** ablation, atrial fibrillation, obstructive sleep apnea, outcomes, pulmonary vein isolation

## Abstract

**Background:**

Obstructive sleep apnea syndrome (OSAS) is common in atrial fibrillation (AF) and may influence AF phenotype and post‐ablation recurrence. We aimed to determine whether documented OSAS identifies a distinct clinical phenotype and whether it is associated with post‐ablation rhythm‐related outcomes.

**Methods:**

We performed a single‐center observational study with two overlapping cohorts. The principal registry cohort comprised 2559 patients with first AF ablation with pulmonary vein isolation (PVI) treated between November/2021 and July/2025. Its primary endpoint was the same‐center rhythm‐intervention endpoint, defined as the first post‐index repeat PVI or electrical cardioversion at the study center. A 7‐day Holter was scheduled 90 days after ablation. The nested active 2022 cohort comprised 666 AF/PVI patients.

**Results:**

Documented OSAS was present in 268 of 2559 registry patients, 10.5%, and in 66 of 652 patients with known OSAS status in the active subset, 10.1%. Documented OSAS was associated with higher BMI, more obesity, hypertension, diabetes, persistent AF, higher triglycerides and liver enzymes, and lower HDL cholesterol. In the registry cohort, the same‐center rhythm‐intervention endpoint occurred in 59/268 patients with documented OSAS (22.0%) and 521/2291 without documented OSAS (22.7%; *p* = 0.788). Documented OSAS was not associated with the registry endpoint after adjustment (OR: 0.87, 95% CI: 0.63–1.21; *p* = 0.411). In the active 2022 cohort, recurrence occurred in 16/32 patients with documented OSAS (50.0%) and 167/302 without documented OSAS (55.3%; *p* = 0.581). Adjusted Cox analysis also showed no independent documented OSAS (HR: 1.08, 95% CI: 0.61–1.94; *p* = 0.786).

**Conclusion:**

Documented OSAS identified an adverse cardiometabolic AF ablation phenotype but was not independently associated with same‐center rhythm interventions.

## Graphical abstract

Documented OSAS identified a higher‐risk cardiometabolic phenotype in patients undergoing AF ablation, including greater obesity, hypertension, diabetes, persistent AF, and adverse lipid markers. However, it did not independently predict post‐ablation rhythm‐related outcomes in either the registry cohort or the actively followed cohort.

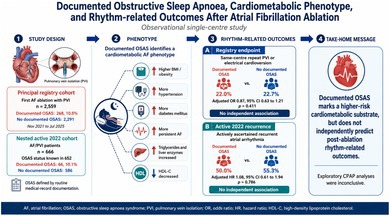

## 1 Introduction

Atrial fibrillation (AF) prevalence and incidence have escalated considerably during the past two decades. They are expected to escalate further, thus constituting one of the most formidable public health threats worldwide [1, 2, 3, 4, 5, 6]. The European Society of Cardiology places ablation within broader AF care pathways rather than as an isolated procedure [7].

Sleep‐disordered breathing is common in AF, but it is often missed in routine care. Recent reviews report wide prevalence estimates that depend heavily on how patients are screened and tested, with many systematically screened AF cohorts showing higher rates than those observed in routine clinical documentation [8, 9]. The 2024 ESC guideline therefore advises against relying only on symptom questionnaires in AF, and diagnostic guidance from the American Academy of Sleep Medicine supports formal testing with polysomnography or home sleep apnea testing in appropriate adults [7, 10].

The biological link between OSAS and AF is plausible. Intermittent hypoxia, intrathoracic pressure swings, autonomic surges, inflammation, and atrial structural and electrical remodeling can all support AF initiation and maintenance [11]. Observational studies and meta‐analyses have often found more recurrence after ablation in patients with OSAS, while CPAP data remain mixed, especially when comparing observational studies with smaller randomized trials [12].

Recent work suggests the association is more nuanced than earlier summaries implied. Outcomes may differ by AF subtype, atrial substrate, and CPAP adherence. A study linked long‐term CPAP adherence in severe OSAS with lower very late recurrence after ablation [10]. A post hoc CABANA analysis further suggested that OSAS treatment status may modify the effectiveness of ablation [13].

Against that background, the present study addresses a practical clinical question. In routine electrophysiology care, documented OSAS in the medical record is examined to determine whether it identifies a higher‐risk ablation phenotype and predicts recurrence, using two definitions: a same‐center hard‐event endpoint and an actively ascertained recurrence endpoint. We hypothesized that documented OSAS would identify a more adverse baseline phenotype, while its association with outcomes could depend on endpoint ascertainment. We also explored whether recorded CPAP use was associated with outcomes among patients with documented OSAS.

## 2 Methods

### 2.1 Design and Setting

We conducted a single‐center observational study of consecutive patients undergoing catheter ablation for AF with pulmonary vein isolation (PVI). The analysis used two overlapping populations with different outcome ascertainment. The principal registry cohort was used to characterize the documented OSAS phenotype and to evaluate the same‐center rhythm intervention after AF/PVI ablation. The nested active follow‐up 2022 cohort was analyzed separately because it had broader active follow‐up and recurrence ascertainment. The active cohort was not an external validation cohort; it was contained within the institutional registry and was used as a complementary active‐follow‐up subset.

The study was reviewed and ethically approved by the institutional review board of the University of Witten/Herdecke (no. S‐192/2023). The reporting of this observational study adhered to the Strengthening the Reporting of Observational Studies in Epidemiology (STROBE) guidelines [14].

### 2.2 Participants

The principal registry cohort included 2559 unique patients treated between 23 November 2021 and 1 July 2025. Patients were eligible if AF was the target arrhythmia and PVI was performed. When more than one eligible AF/PVI procedure was present for a patient, the first ablation during the study period was retained as the index procedure; subsequent cardioversions or repeat ablations were retained as potential outcome events. The active follow‐up cohort included 666 patients who underwent AF/PVI ablation in 2022. All 666 active‐cohort patients were present in the raw institutional registry, confirming that the active cohort was nested within the registry rather than independent of it.

### 2.3 Clinical Data and Exposure Definition

Baseline data were abstracted from procedure reports, discharge summaries, diagnosis lists, medication lists, electrocardiograms, laboratory results, and echocardiographic information when available. Comorbidities were assigned from documented clinical diagnoses rather than administrative billing codes. Clinical variables included age, sex, body mass index (BMI), AF phenotype, hypertension, diabetes mellitus, heart failure, coronary artery disease (CAD), prior stroke or transient ischemic attack (TIA), chronic kidney disease (CKD), peripheral vascular disease, valvular heart disease or surgery, cardiomyopathy, thyroid disease, smoking history, left ventricular ejection fraction (LVEF) when available, and relevant medication classes at the time of ablation, including antiarrhythmic drugs (AADs).

The primary exposure was documented OSAS in routine clinical care. Documented OSAS was defined by medical‐record documentation of sleep apnea, obstructive sleep apnea, OSAS, or equivalent terminology at the time of ablation. Patients without such documentation were classified as having no documented OSAS. This reference group should not be interpreted as patients objectively free of sleep‐disordered breathing, because systematic sleep testing was not performed. Among patients with documented OSAS, CPAP therapy was analyzed as an exploratory subgroup exposure based on documented CPAP or CPAP mask use. OSAS severity, apnea‐hypopnea index, nocturnal hypoxemia, CPAP adherence, treatment duration, and residual respiratory burden were unavailable.

### 2.4 Ablation Procedures and Procedural Variables

All procedures were performed in an experienced electrophysiology laboratory in accordance with contemporary clinical practice. PVI was the procedural endpoint. Cryoballoon or radiofrequency ablation, and any additional lesion set, were chosen by the treating electrophysiologist according to AF type, prior ablation history, concomitant atrial flutter, substrate, and intra‐procedural findings. Additional ablation could include cavotricuspid isthmus ablation, atrial flutter ablation, left atrial line ablation, or substrate modification when clinically indicated.

Cryoblloon procedural parameters were available in the active 2022 subset and were analyzed by OSAS status. For each pulmonary vein, the number of freezes/applications, freeze duration, and nadir temperature were summarized for the left superior pulmonary vein (LSPV), left inferior pulmonary vein (LIPV), right superior pulmonary vein (RSPV), and right inferior pulmonary vein (RIPV).

### 2.5 Outcomes

All patients were scheduled for 7‐day Holter monitoring approximately 90 days after ablation, followed by clinical review either in person or by telephone. Recurrence documented during this standardized post‐blanking Holter assessment or follow‐up consultation was recorded when available. Longitudinal outcome ascertainment then differed by cohort.

In the principal registry cohort, the primary endpoint was the same‐center rhythm‐intervention endpoint, defined as the first post‐index repeat PVI or electrical cardioversion performed at the study center. This endpoint was selected because these events could be verified with high certainty in the retrospective registry and reflect clinically actionable rhythm‐related care after ablation. The endpoint was not intended to capture all recurrent AF. It may miss recurrent AF managed conservatively, recurrent AF accepted as permanent, recurrence treated only with antiarrhythmic drug therapy or outpatient follow‐up, asymptomatic or low‐burden recurrence, and recurrence documented or treated outside the study center. All patients underwent standardized 7‐day Holter monitoring approximately 90 days after ablation followed by clinical review; thereafter, long‐term registry ascertainment was based on verifiable same‐center repeat PVI or electrical cardioversion. The nested active 2022 cohort was therefore analyzed separately because it had broader recurrence ascertainment through direct follow‐up, chart review, available external reports, ECG/Holter documentation, cardioversion, repeat ablation, and documented recurrence‐related care.

Registry sensitivity analyses applied a 90‐day blanking period and one‐year outcome windows among patients with at least 365 days of potential administrative observation. In the active 2022 cohort, recurrence was defined as any documented recurrent atrial arrhythmia after the index ablation based on direct patient contact, chart review, available external records, electrocardiography/Holter documentation, cardioversion, repeat ablation, or documented recurrence‐related care. Patients without documented recurrence were censored at the last available follow‐up date. Active time‐to‐event analyses were restricted to patients with valid event or censoring dates.

### 2.6 Statistical Analysis

Continuous variables are summarized as mean ± standard deviation (SD) or median (interquartile range [IQR]), depending on distribution. Categorical variables are summarized as *n*/*N* (%). Two‐group comparisons used Welch *t*‐tests or Mann–Whitney *U*‐tests for continuous variables and chi‐squared or Fisher exact tests for categorical variables, as appropriate. Laboratory‐panel *p*‐values were corrected with the Benjamini–Hochberg false‐discovery‐rate (FDR) method.

Baseline comparisons characterized the documented OSAS phenotype. Logistic regression was the primary modeling approach for the binary registry same‐center rhythm‐intervention endpoint because complete patient‐level long‐term rhythm‐monitoring and external event capture were unavailable. Registry Kaplan–Meier and Cox proportional hazards analyses were reported as supplementary time‐to‐event analyses using administrative censoring. In the nested active 2022 cohort, Kaplan–Meier and Cox proportional hazards analyses were used for actively ascertained recurrence among patients with valid event or censoring dates. Cox models are reported as HRs with 95% CIs. Registry adjusted models included documented OSAS, age, sex, BMI, persistent AF, hypertension, diabetes mellitus, heart failure, CAD, and CKD when applicable. Active‐cohort adjusted models included documented OSAS, age, sex, persistent AF, hypertension, diabetes mellitus, heart failure, CAD, and CKD. Missing data were not imputed; analyses used available cases. Two‐sided *p* < 0.05 was considered statistically significant.

## 3 Results

### 3.1 Study Population and Cohort Architecture

The raw registry contained 5817 procedure records and 397 columns. After selecting AF procedures with PVI documentation and retaining the first eligible AF/PVI procedure per patient, the final principal registry cohort comprised 2559 patients. Documented OSAS was present in 268 patients (10.5%), and 2291 patients had no documented OSAS. Among patients with documented OSAS, CPAP therapy was recorded in 117/268 (43.7%).

**FIGURE 1:**
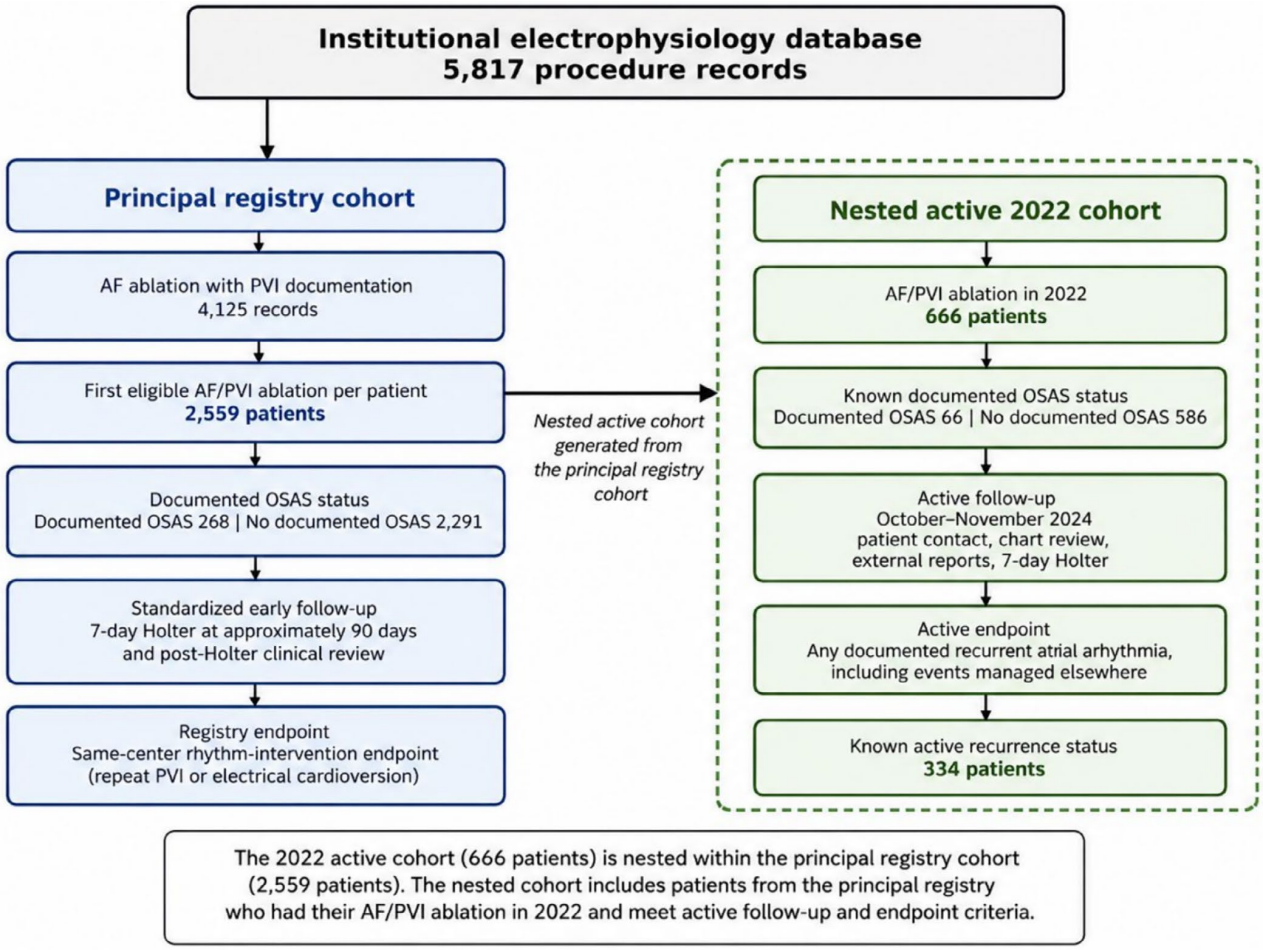
Cohort flow and analytic architecture. The active 2022 cohort was nested within the principal registry and was analyzed separately because recurrence ascertainment differed.

The nested active 2022 follow‐up cohort comprised 666 AF/PVI patients. OSAS status was known in 652 patients, including 66 patients with documented OSAS (10.1%) and 586 without documented OSAS. CPAP therapy was recorded in 30/66 patients with documented OSAS (45.5%). Active recurrence status was known in 334/652 patients with known OSAS status, and 309 patients contributed usable active time‐to‐event data. Because all active‐cohort patients were present in the raw registry, the cohorts were treated as overlapping cohorts with different recurrence ascertainment rather than independent cohorts. Figure 1 provides an overview of the cohort.

### 3.2 Baseline Phenotype in the Principal Registry Cohort

Documented OSAS identified a clinically coherent cardiometabolic AF ablation phenotype (Table 1). Compared with patients without documented OSAS, patients with documented OSAS had higher BMI, more obesity, lower female proportion, more hypertension, more diabetes mellitus, and more persistent AF. CAD was numerically more frequent in the documented OSAS group, while heart failure, prior stroke/TIA, CKD, and LVEF were not significantly different in available‐case analyses.

**TABLE 1:** Principal registry cohort baseline characteristics by documented OSAS status.

Characteristic	No documented OSAS	Documented OSAS	*p*
Age, years	68.0 [60.0, 75.0]	66.0 [60.8, 73.0]	0.063
BMI, kg/m^2^	26.0 [24.0, 29.0] (*n* = 2154)	29.0 [26.0, 32.0] (*n* = 260)	< 0.001
Female sex	867/2291 (37.8%)	52/268 (19.4%)	< 0.001
Obesity (BMI ≥ 30 kg/m^2^)	528/2154 (24.5%)	126/260 (48.5%)	< 0.001
Paroxysmal AF	473/2291 (20.6%)	50/268 (18.7%)	0.445
Persistent AF	921/2291 (40.2%)	128/268 (47.8%)	0.017
AF type unknown/not documented	897/2291 (39.2%)	90/268 (33.6%)	0.076
Heart failure	165/2291 (7.2%)	19/268 (7.1%)	0.946
Hypertension	1274/2291 (55.6%)	184/268 (68.7%)	< 0.001
Diabetes mellitus	230/2291 (10.0%)	44/268 (16.4%)	0.001
Stroke/TIA	151/2291 (6.6%)	21/268 (7.8%)	0.441
Coronary artery disease	607/2291 (26.5%)	86/268 (32.1%)	0.051
Peripheral vascular disease	33/2291 (1.4%)	4/268 (1.5%)	0.792
Chronic kidney disease	212/2291 (9.3%)	33/268 (12.3%)	0.107
Hyperlipidemia	738/2291 (32.2%)	92/268 (34.3%)	0.484
Smoking history	267/2291 (11.7%)	36/268 (13.4%)	0.394
Thyroid disease	520/2291 (22.7%)	57/268 (21.3%)	0.596
Rheumatic disease	51/2291 (2.2%)	13/268 (4.9%)	0.009
Device present	163/2291 (7.1%)	14/268 (5.2%)	0.248
Any cardiomyopathy	101/2291 (4.4%)	17/268 (6.3%)	0.153
Any valve disease/surgery	676/2291 (29.5%)	85/268 (31.7%)	0.454
LVEF, %, available‐case registry subset	50.0 [40.0, 58.0] (*n* = 353)	52.0 [40.0, 56.0] (*n* = 45)	0.884
Beta‐blocker therapy	1337/2203 (60.7%)	135/264 (51.1%)	0.003
Any oral anticoagulation	1663/2291 (72.6%)	171/268 (63.8%)	0.003
Any class I/III AAD	634/2291 (27.7%)	61/268 (22.8%)	0.087
SGLT2 inhibitor	157/2203 (7.1%)	30/264 (11.4%)	0.014
GLP1 agonist	26/2203 (1.2%)	2/264 (0.8%)	0.762
Calcium‐channel blocker	306/2203 (13.9%)	52/264 (19.7%)	0.011
ACE inhibitor or ARB/ARNI	791/2203 (35.9%)	102/264 (38.6%)	0.383

*Note:* Values are median [IQR], mean ± SD, or *n*/*N* (%). LVEF was available only in a registry subset and is reported as available‐case data.

Abbreviations: AAD, antiarrhythmic drug; AF, atrial fibrillation; BMI, body mass index; LVEF, left ventricular ejection fraction; TIA, transient ischemic attack.

The laboratory profile supported a broader cardiometabolic phenotype (Table 2). After FDR correction, documented OSAS was associated with higher leukocytes, erythrocytes, hemoglobin, hematocrit, alanine aminotransferase/glutamate‐pyruvate transaminase (ALT/GPT), gamma‐glutamyl transferase (GGT), and triglycerides, and with lower HDL‐C, total cholesterol, and potassium.

**TABLE 2:** Selected laboratory profile in the principal registry cohort by documented OSAS status.

Laboratory variable	No documented OSAS	Documented OSAS	*p* value; FDR *q*
Leukocytes	6.90 [5.80, 8.10] (*n* = 1764)	7.20 [6.10, 8.50] (*n* = 215)	0.006; *q* = 0.024
Hemoglobin	14.4 [13.5, 15.3] (*n* = 1761)	14.6 [13.8, 15.6] (*n* = 215)	0.004; *q* = 0.020
Hematocrit	43.0 [41.0, 45.0] (*n* = 1761)	44.0 [41.0, 47.0] (*n* = 215)	0.003; *q* = 0.020
Creatinine	0.96 [0.83, 1.10] (*n* = 1758)	0.97 [0.85, 1.12] (*n* = 215)	0.259; *q* = 0.428
eGFR CKD‐EPI	75.0 [62.0, 87.0] (*n* = 1758)	77.0 [65.0, 90.0] (*n* = 215)	0.028; *q* = 0.076
ALT/GPT	23.0 [18.0, 30.5] (*n* = 1679)	26.0 [20.5, 32.0] (*n* = 207)	< 0.001; *q* = 0.009
GGT	26.0 [18.0, 42.0] (*n* = 1688)	31.0 [21.0, 45.0] (*n* = 207)	0.001; *q* = 0.012
Total cholesterol	187 [154, 216] (*n* = 1664)	180 [140, 210] (*n* = 205)	0.009; *q* = 0.030
Triglycerides	111 [82.0, 154] (*n* = 1677)	125 [90.5, 175] (*n* = 207)	0.002; *q* = 0.016
HDL‐C	56.0 [47.0, 68.0] (*n* = 1661)	50.5 [41.0, 60.2] (*n* = 204)	< 0.001; *q* < 0.001
LDL‐C	111 [83.0, 140] (*n* = 1661)	106 [72.8, 133] (*n* = 204)	0.097; *q* = 0.187
Potassium	4.40 [4.20, 4.60] (*n* = 1750)	4.30 [4.10, 4.50] (*n* = 214)	0.004; *q* = 0.020
HbA1c	37.5 [34.5, 41.0] (*n* = 46)	43.5 [40.8, 49.5] (*n* = 8)	0.036; *q* = 0.091

*Note:* Values are median [IQR]. FDR *q* values correct for the laboratory panel.

Abbreviations: ALT/GPT, alanine aminotransferase/glutamate‐pyruvate transaminase; eGFR, estimated glomerular filtration rate; GGT, gamma‐glutamyl transferase; HbA1c, glycated hemoglobin; HDL‐C. high‐density lipoprotein cholesterol; LDL‐C, low‐density lipoprotein cholesterol.

### 3.3 Registry Same‐Center Rhythm‐Intervention Endpoint

Registry procedural text‐derived characteristics were broadly balanced by documented OSAS status. Cryoballoon PVI text was present in 195/268 patients with documented OSAS (72.8%) and 1751/2291 without documented OSAS (76.4%; *p* = 0.183). Radiofrequency/3D mapping PVI text was present in 89/268 (33.2%) versus 679/2291 (29.6%; *p* = 0.227), and additional CTI/isthmus/atrial flutter ablation text in 30/268 (11.2%) versus 237/2291 (10.3%; *p* = 0.667).

The same‐center rhythm‐intervention endpoint occurred in 59/268 patients with documented OSAS (22.0%) and 521/2291 without documented OSAS (22.7%; *p* = 0.788) (Table 3). Repeat PVI after the index procedure occurred in 38/268 (14.2%) versus 317/2291 (13.8%; *p* = 0.878). Electrical cardioversion after the index procedure occurred in 41/268 (15.3%) versus 349/2291 (15.2%; *p* = 0.978). After a 90‐day blanking period, the endpoint occurred in 54/268 patients with documented OSAS (20.1%) and 460/2291 without documented OSAS (20.1%; *p* = 0.978). One‐year sensitivity analyses were similarly neutral.

**TABLE 3:** Principal registry same‐center rhythm‐intervention endpoint components and sensitivity analyses.

Endpoint component/sensitivity analysis	No documented OSAS	Documented OSAS	*p*
Any post‐index same‐center repeat PVI/electrical cardioversion	521/2291 (22.7%)	59/268 (22.0%)	0.788
Repeat PVI after index procedure	317/2291 (13.8%)	38/268 (14.2%)	0.878
Electrical cardioversion after index procedure	349/2291 (15.2%)	41/268 (15.3%)	0.978
Any event after 90‐day blanking	460/2291 (20.1%)	54/268 (20.1%)	0.978
Repeat PVI after 90‐day blanking	312/2291 (13.6%)	38/268 (14.2%)	0.800
Cardioversion after 90‐day blanking	271/2291 (11.8%)	31/268 (11.6%)	0.900
Any event within 1 year	308/1972 (15.6%)	35/215 (16.3%)	0.800
Any event from day 91 to day 365	246/1972 (12.5%)	32/215 (14.9%)	0.314

*Note:* The registry endpoint was the first post‐index repeat PVI or electrical cardioversion performed at the study center.

### 3.4 Adjusted Registry and Active‐Cohort Models

In the principal registry cohort, documented OSAS was not associated with the same‐center rhythm‐intervention endpoint in unadjusted or adjusted analyses (Table 4). The adjusted logistic OR was 0.87 (95% CI 0.63–1.21; *p* = 0.411). Supplementary Cox modeling with administrative censoring likewise showed no association for documented OSAS (adjusted HR 0.94, 95% CI 0.71–1.25; *p* = 0.681). Persistent AF was strongly associated with the endpoint in the adjusted registry models.

**TABLE 4:** Multivariable and sensitivity models.

Dataset/model	Variable	Effect estimate	*p*	*N*/events
Registry unadjusted logistic: same‐center rhythm‐intervention endpoint	Documented OSAS vs. no documented OSAS	OR 0.96 (95% CI 0.71–1.30)	0.789	2559/580
Registry adjusted logistic: same‐center rhythm‐intervention endpoint	Documented OSAS vs. no documented OSAS	OR 0.87 (95% CI 0.63–1.21)	0.411	2414/547
Registry adjusted logistic: same‐center rhythm‐intervention endpoint	Persistent AF	OR 1.97 (95% CI 1.61–2.42)	< 0.001	2414/547
Registry supplementary adjusted Cox: same‐center rhythm‐intervention endpoint	Documented OSAS vs. no documented OSAS	HR 0.94 (95% CI 0.71–1.25)	0.681	2414/547
Registry supplementary adjusted Cox: after 90‐day blanking	Documented OSAS vs. no documented OSAS	HR 1.05 (95% CI 0.78–1.41)	0.743	2414/483
Active 2022 adjusted Cox: actively ascertained recurrence	Documented OSAS vs. no documented OSAS	HR 1.08 (95% CI 0.61–1.94)	0.786	293/154
Active 2022 adjusted Cox: actively ascertained recurrence	Female sex	HR 1.50 (95% CI 1.08–2.07)	0.016	293/154
Active 2022 adjusted Cox: actively ascertained recurrence	Persistent AF	HR 1.47 (95% CI 1.05–2.05)	0.024	293/154

*Note:* Registry logistic regression was the primary model for the binary same‐center rhythm‐intervention endpoint. Registry Cox analyses used administrative censoring and are supplementary. Active‐cohort Cox analyses used actively ascertained recurrence and valid event or censoring dates.

Abbreviations: CI, confidence interval; HR, hazard ratio; OR, odds ratio.

**FIGURE 2:**
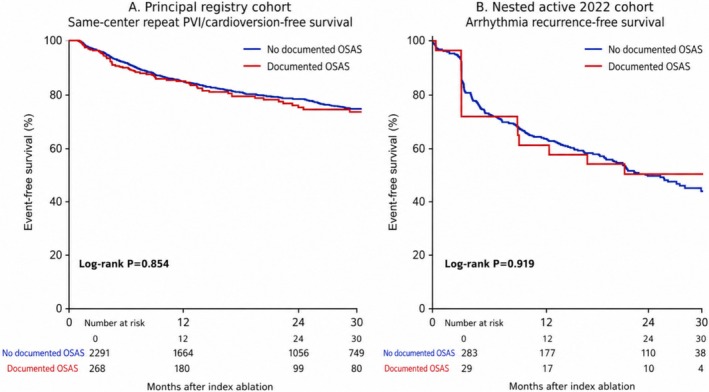
Kaplan–Meier curves by documented OSAS status in the principal registry cohort and the nested active 2022 follow‐up cohort. Panel A shows freedom from same‐center repeat PVI or electrical cardioversion. Panel B shows freedom from actively ascertained recurrent atrial arrhythmia. *p* values are from log‐rank tests. Registry time‐to‐event analysis used administrative censoring and is supplementary to the binary registry model.

Kaplan–Meier analysis (Figure 2) showed no separation of event‐free survival by documented OSAS status in the registry cohort (log‐rank *p* = 0.854). In the active 2022 cohort, recurrence‐free survival also did not differ by documented OSAS status (log‐rank *p* = 0.83). Active time‐to‐event analysis included 309 patients with known OSAS status and valid event/censoring dates.

### 3.5 Nested Active 2022 Cohort

The active 2022 cohort reproduced the principal baseline pattern in a smaller, actively followed subset (Table 5). Patients with documented OSAS were less often female and had more hypertension and diabetes mellitus. LVEF was similar by OSAS status. Available‐case left atrial measurements were numerically higher among patients with documented OSAS, but these exploratory echocardiographic substrate variables were not statistically significant.

**TABLE 5:** Baseline characteristics of the nested active 2022 cohort by documented OSAS status.

Characteristics	No documented OSAS	Documented OSAS	*p*
Age, years	67.5 [59.2, 74.7]	66.0 [59.8, 72.5]	0.381
BMI, kg/m^2^	26.0 [24.0, 30.0] (*n* = 566)	29.0 [26.0, 32.0] (*n* = 66)	< 0.001
Female sex	230/586 (39.2%)	14/66 (21.2%)	0.005
Obesity, BMI ≥ 30 kg/m^2^	148/566 (26.1%)	29/66 (43.9%)	0.004
Paroxysmal AF	347/586 (59.2%)	38/66 (57.6%)	0.793
Persistent AF	238/586 (40.6%)	28/66 (42.4%)	0.793
AF type unknown/not documented	1/586 (0.2%)	0/66 (0.0%)	1.000
Previous PVI/AF ablation	160/586 (27.3%)	21/66 (31.8%)	0.469
Previous cardioversion	315/586 (53.8%)	36/66 (54.5%)	1.000
CHA_2_DS_2_‐VASc score	1.0 [1.0, 3.0]	2.0 [1.0, 3.0]	0.247
LVEF, %	60.0 [55.0, 60.0] (*n* = 554)	60.0 [55.0, 60.0] (*n* = 64)	0.524
LVEF < 50%	36/554 (6.5%)	7/64 (10.9%)	0.193
Left atrial volume, available‐case	163.4 [135.0, 187.4] (*n* = 103)	176.1 [151.8, 218.0] (*n* = 17)	0.107
Left atrial surface, available‐case	232.7 [205.4, 253.2] (*n* = 103)	262.0 [232.0, 274.1] (*n* = 17)	0.054
Heart failure	46/586 (7.8%)	9/66 (13.6%)	0.156
Hypertension	373/586 (63.7%)	52/66 (78.8%)	0.014
Diabetes mellitus	65/586 (11.1%)	13/66 (19.7%)	0.047
Stroke/TIA	50/586 (8.5%)	6/66 (9.1%)	0.818
Coronary artery disease	138/585 (23.6%)	21/66 (31.8%)	0.173
Peripheral vascular disease	15/586 (2.6%)	2/66 (3.0%)	0.687
Chronic kidney disease	63/586 (10.8%)	8/66 (12.1%)	0.680
Hyperlipidemia	213/586 (36.3%)	26/66 (39.4%)	0.686
Smoking history	64/586 (10.9%)	7/66 (10.6%)	1.000
Thyroid disease	116/586 (19.8%)	18/66 (27.3%)	0.152
Rheumatic disease	26/586 (4.4%)	9/66 (13.6%)	0.005
Device present	35/586 (6.0%)	4/66 (6.1%)	1.000
Any cardiomyopathy	59/586 (10.1%)	10/66 (15.2%)	0.206
Any valve disease/surgery	173/586 (29.5%)	21/66 (31.8%)	0.673
Oral anticoagulation at baseline	554/586 (94.5%)	64/66 (97.0%)	0.564
Any class I/III AAD at baseline	226/586 (38.6%)	19/66 (28.8%)	0.140
Beta‐blocker at baseline	445/586 (75.9%)	50/66 (75.8%)	1.000
Statin at baseline	256/586 (43.7%)	31/66 (47.0%)	0.695
SGLT2 inhibitor	26/585 (4.4%)	6/65 (9.2%)	0.121
GLP1 agonist	8/586 (1.4%)	0/66 (0.0%)	1.000
Calcium‐channel blocker	120/586 (20.5%)	22/66 (33.3%)	0.026
ACE inhibitor or ARB/ARNI	318/586 (54.3%)	45/66 (68.2%)	0.036

*Note:* Values are median [IQR] or *n*/*N* (%). BMI was available through exact patient/procedure‐date mapping to the registry. LVEF was reported as valid available‐case data; EF values coded as 0 were treated as missing.

Abbreviations: AAD, antiarrhythmic drug; AF, atrial fibrillation; BMI, body mass index; GLP1, glucagon‐like peptide‐1; LVEF, left ventricular ejection fraction; SGLT2, sodium‐glucose cotransporter 2; TIA, transient ischemic attack.

In the active 2022 cohort, recurrence among patients with known recurrence status occurred in 16/32 patients with documented OSAS (50.0%) and 167/302 without documented OSAS (55.3%; *p* = 0.581) (Table 6). The adjusted active‐cohort Cox model did not show an independent documented OSAS association (HR 1.08, 95% CI 0.61–1.94; *p* = 0.786). Female sex and persistent AF were associated with recurrence in this model.

**TABLE 6:** Nested active 2022 cohort follow‐up outcomes by documented OSAS status.

Characteristics	No documented OSAS	Documented OSAS	*p*/estimate
Known recurrence status	302/586 (51.5%)	32/66 (48.5%)	0.697
Recurrence among those with known status	167/302 (55.3%)	16/32 (50.0%)	0.581
Usable for active time‐to‐event analysis	281/586 (48.0%)	28/66 (42.4%)	0.436
Observation time to recurrence/censor, months	20.6 [5.3–26.6]	19.9 [3.1–25.3]	0.664
Estimated 12‐month freedom from recurrence	64.0%	60.7%	Descriptive KM estimate
Estimated 24‐month freedom from recurrence	50.4%	53.1%	Descriptive KM estimate
Log‐rank test	Reference	Documented OSAS	*p* = 0.83
Recurrence during blanking period	85/358 (23.7%)	10/40 (25.0%)	0.846
Redo ablation during follow‐up	66/265 (24.9%)	4/26 (15.4%)	0.343
Cardioversion required during follow‐up	29/354 (8.2%)	4/40 (10.0%)	0.761
Hospitalization required during active follow‐up	39/354 (11.0%)	4/40 (10.0%)	1.000

*Note:* Active recurrence was ascertained by direct follow‐up, chart review, available external records, electrocardiography/Holter documentation, cardioversion, repeat ablation, or documented recurrence‐related care. These outcomes were not pooled with the registry same‐center rhythm‐intervention endpoint.

### 3.6 Exploratory CPAP Analyses

CPAP analyses were exploratory because CPAP was recorded only as a binary treatment label and did not capture OSAS severity, adherence, treatment duration, residual apnea‐hypopnea index, or residual nocturnal hypoxemia. In the registry OSAS subgroup, same‐center rhythm‐intervention events were numerically more frequent among patients with recorded CPAP use, but this difference was not statistically significant. In the active 2022 OSAS subgroup, recurrence was numerically lower among CPAP users, but the sample size was small and the result was inconclusive (Table 7).

**TABLE 7:** Exploratory CPAP analyses among patients with documented OSAS.

Cohort/endpoint	Documented OSAS without CPAP	Documented OSAS with CPAP	Effect estimate/*p*
Registry same‐center rhythm‐intervention endpoint	29/151 (19.2%)	30/117 (25.6%)	Adjusted HR 1.26 (95% CI 0.75–2.13); *p* = 0.380
Registry endpoint after 90‐day blanking	27/151 (17.9%)	27/117 (23.1%)	Descriptive *p* = 0.293
Active 2022 recurrence among known statuses	10/17 (58.8%)	6/15 (40.0%)	Unadjusted HR 0.55 (95% CI 0.18–1.67); *p* = 0.290
Active 2022 blanking‐period recurrence	8/21 (38.1%)	2/19 (10.5%)	Descriptive *p* = 0.069

*Note:* CPAP status was captured as a binary documentation variable and should not be interpreted as CPAP adherence, treatment duration, or OSAS severity.

### 3.7 Active 2022 Subset: Cryoballoon Procedural Parameters by OSAS Status

Cryoballoon procedural parameters were available for cryoballoon procedures in the active 2022 dataset. There were no statistically significant differences by OSAS status in vein‐specific number of freezes/applications, freeze duration, or nadir temperature (Table 8).

**TABLE 8:** Active 2022 subset cryoballoon procedural parameters by documented OSAS status.

Cryoballoon parameter	No documented OSAS	Documented OSAS	*p*	Available *N*
LSPV: number of freezes/applications	1 [1 to 1]	1 [1 to 1]	0.793	417
LSPV: freeze time, seconds	240 [240 to 240]	240 [240 to 240]	0.223	415
LSPV: nadir temperature, °C	−52 [−55 to −48]	−53 [−56 to −50]	0.105	413
LIPV: number of freezes/applications	1 [1 to 2]	1 [1 to 2]	0.184	417
LIPV: freeze time, seconds	240 [240 to 240]	240 [240 to 240]	0.561	415
LIPV: nadir temperature, °C	−48 [−51 to −45]	−49 [−53 to −45]	0.163	413
RSPV: number of freezes/applications	1 [1 to 2]	1 [1 to 2]	0.527	432
RSPV: freeze time, seconds	240 [180 to 240]	240 [180 to 240]	0.320	429
RSPV: nadir temperature, °C	−53 [−57 to −48]	−53 [−58 to −50]	0.402	428
RIPV: number of freezes/applications	1 [1 to 2]	1 [1 to 2]	0.850	430
RIPV: freeze time, seconds	240 [232 to 240]	240 [240 to 240]	0.601	428
RIPV: nadir temperature, °C	‐50 [−53 to −46]	−51 [−56 to −46]	0.357	424

*Note:* Cryoballoon parameters were available only for cryoballoon procedures in the active 2022 dataset.

Abbreviations: LIPV, left inferior pulmonary vein; LSPV, left superior pulmonary vsein; RIPV, right inferior pulmonary vein; RSPV, right superior pulmonary vein.

## 4 Discussion

In this single‐center cohort of patients undergoing AF ablation with PVI, documented OSAS identified a reproducible adverse cardiometabolic phenotype but was not independently associated with rhythm‐related outcomes. Patients with documented OSAS had higher BMI, more obesity, hypertension, diabetes mellitus, persistent AF, hypertriglyceridemia, lower HDL‐C, and higher liver enzymes. Despite this higher‐risk baseline profile, the same‐center rhythm‐intervention endpoint was similar in patients with and without documented OSAS in the principal registry cohort, including after a 90‐day blanking period. The nested active follow‐up cohort, analyzed separately with broader recurrence ascertainment, showed the same absence of an independent documented OSAS signal.

The distinction between documented OSAS as a substrate marker and documented OSAS as an independent outcome predictor is clinically important. OSA is mechanistically linked to AF through intermittent hypoxemia, intrathoracic pressure swings, autonomic activation, oxidative stress, systemic inflammation, endothelial dysfunction, and atrial structural and electrical remodeling [11, 15, 16, 17]. These mechanisms do not act in isolation. They interact with obesity, hypertension, insulin resistance, dyslipidemia, and epicardial or visceral adiposity, all of which may promote atrial stretch, fibrosis, conduction heterogeneity, and AF maintenance [18]. The phenotype observed here is therefore biologically plausible: documented OSAS marked a broader metabolic and vascular AF substrate rather than an isolated exposure.

This distinction between OSA as a substrate marker and as an independent predictor of recurrence is important. AF initiation is frequently associated with ectopic activity originating from the pulmonary veins, providing the mechanistic rationale for PVI [19]. OSA, in contrast, may contribute both to triggers and to atrial vulnerability, but its longer‐term effect is likely mediated through atrial remodeling, cardiometabolic disease, and autonomic imbalance [20]. Therefore, a mechanistic association between OSA and AF does not necessarily imply a binary routine‐record OSAS label will independently predict recurrence after PVI. If pulmonary‐vein trigger activity is effectively isolated, recurrent atrial arrhythmia may depend less on the presence of a documented OSAS diagnosis and more on the cumulative burden of atrial substrate, AF phenotype, obesity, hypertension, diabetes, left atrial remodeling, and lesion durability.

The present findings differ from several earlier observational studies and meta‐analyses that reported higher recurrence after ablation in patients with OSA and lower recurrence among CPAP‐treated patients [21, 22, 23]. However, the earlier literature is heterogeneous in OSA definitions, sleep testing, rhythm monitoring, ablation strategies, AF phenotypes, treatment adherence, and adjustment for cardiometabolic confounding. Some influential studies were performed in RF‐dominant cohorts and focused on patients with untreated or more severe OSAS [24, 25]. More recent evidence has been less uniform. In the randomized trial by Hunt et al., patients with paroxysmal AF and OSAS were randomized to CPAP or standard care after PVI and monitored with implantable loop recorders; CPAP markedly reduced AHI, but AF recurrence and AF burden were not further reduced compared with standard care [12]. Recent analyses have also emphasized heterogeneity by AF phenotype, OSAS severity, and treatment adherence rather than a uniform OSAS effect across all ablation populations [10, 26, 27].

A procedural explanation should also be considered. The present cohort was predominantly treated with cryoballoon PVI. Randomized studies have shown that cryoballoon PVI is as effective as RF ablation in paroxysmal AF and is an established contemporary PVI strategy [28, 29]. In the active 2022 subset of the present study, vein‐specific cryoballoon parameters, including the number of applications, freeze duration, and nadir temperature, did not differ significantly by documented OSAS status. This does not prove equivalent lesion durability because systematic remapping was unavailable. Nevertheless, it makes a major measurable OSAS‐related difference in cryoballoon application delivery unlikely. It is therefore plausible, although hypothesis‐generating, that a reproducible single‐shot PVI strategy may attenuate some procedural variability that could have influenced outcomes in older or RF‐dominant cohorts. This interpretation should be approached with caution, as the study was not designed to specifically compare cryoballoon and RF ablation, and pulmonary vein reconnection was not systematically assessed.

The low prevalence of documented OSAS in this cohort is also central to interpretation. Documented OSAS was present in approximately 10% of patients, which is substantially lower than rates reported in systematically screened AF cohorts [8, 9]. This discrepancy almost certainly reflects under‐ascertainment in routine clinical records rather than the true absence of sleep‐disordered breathing. Current diagnostic guidance supports formal testing with polysomnography or technically adequate home sleep apnea testing when OSAS is suspected, and the 2024 ESC AF guideline specifically cautions against relying only on symptom‐based questionnaires in AF populations [7, 30]. The present study, therefore, evaluates the prognostic value of documented OSAS in routine electrophysiology practice, not the prognostic value of systematically measured OSAS burden. Undiagnosed OSAS among patients classified as non‐OSAS would bias associations toward the null. In addition, the available OSAS variable did not capture AHI, nocturnal hypoxemia, hypoxic burden, arousal frequency, sleep fragmentation, OSAS phenotype, or treatment response, all of which may be more biologically informative than a binary diagnosis.

The CPAP findings should be interpreted as exploratory and not as evidence for or against CPAP efficacy. In the principal registry cohort, CPAP users had numerically more same‐center hard events, whereas in the active subset, CPAP users had numerically lower recurrence and fewer blanking‐period recurrences. These opposite directions are compatible with confounding by indication and limited power. Patients prescribed CPAP may have had more severe or clinically apparent OSAS, while the active subset was small and unable to support definitive treatment‐effect inference. Importantly, CPAP was captured only as a documented treatment label, without objective adherence, nightly use, residual AHI, treatment duration before ablation, or residual nocturnal hypoxaemia. This limitation is important because recent work suggests that CPAP benefit may depend on sustained adherence and OSAS severity. Nalliah et al. reported evidence that CPAP may reverse aspects of atrial remodeling in AF patients with OSAS [31], while Tanaka et al. found that long‐term CPAP adherence in severe OSAS was associated with lower very late recurrence after AF ablation [10]. A post hoc CABANA analysis also suggested that OSAS treatment status may modify the observed effectiveness of catheter ablation [13]. These data support the view that CPAP prescription alone is an inadequate exposure variable for ablation‐outcome research.

The endpoint architecture of this study provides a useful methodological insight. The principal registry endpoint captured same‐center repeat PVI or electrical cardioversion, which is clinically meaningful and highly specific for actionable recurrence, but it did not capture all rhythm‐documented AF, asymptomatic episodes, conservative outpatient management, or events treated outside the study center. The nested active follow‐up cohort partly addressed this limitation through broader ascertainment. The absence of an OSAS signal in both frameworks strengthens the conclusion that documented OSAS was not a robust predictor of recurrence in this dataset. At the same time, neither endpoint is equivalent to continuous rhythm monitoring; therefore, small differences in AF burden or subclinical recurrence cannot be excluded.

These findings have practical implications. Documented OSAS should not be used as a stand‐alone marker of expected PVI failure or as a reason to discourage ablation when otherwise clinically indicated. Rather, it should prompt structured risk‐factor assessment and management. This interpretation is consistent with contemporary AF care pathways, which place rhythm control within a broader framework of comorbidity management, including weight reduction, blood‐pressure control, diabetes management, alcohol reduction, physical activity, and evaluation for unrecognized or undertreated sleep‐disordered breathing when clinically suspected [32]. In clinical practice, the more relevant question may not be whether OSAS is recorded, but whether the patient has unrecognized or inadequately treated sleep‐disordered breathing, persistent nocturnal hypoxemia, obesity‐related atrial substrate, and modifiable cardiometabolic risk.

## 5 Limitations

The study has limitations that should temper interpretation. OSAS was defined from routine medical documentation rather than systematic sleep testing, and true OSA prevalence was likely underestimated. OSA severity, AHI, oxygen desaturation burden, arousal burden, sleep‐study type, and CPAP adherence were unavailable. Residual confounding is possible because OSAS is closely linked to obesity, hypertension, diabetes, persistent AF, and other comorbidities. The registry endpoint captured the same‐center repeat intervention rather than continuous rhythm‐detected recurrence. The active 2022 cohort had broader recurrence ascertainment but was smaller and nested within the registry rather than independent. LVEF and left atrial measurements were incompletely available in the registry, and cryoballoon procedural parameters were available only in the active subset. Finally, lesion durability and pulmonary‐vein reconnection were not systematically assessed.

## 6 Conclusion

Documented OSAS identified an adverse cardiometabolic phenotype among patients undergoing AF/PVI ablation but did not independently predict same‐center rhythm interventions in the principal registry cohort or actively ascertained recurrent atrial arrhythmia in the nested active cohort. These results challenge a simple model in which any recorded OSAS diagnosis automatically translates into a higher post‐PVI recurrence risk. Instead, they support a more clinically useful interpretation: routine‐record OSAS is a marker of cardiometabolic AF substrate, while recurrence after contemporary PVI is likely determined by a broader interaction among atrial substrate, AF phenotype, procedural durability, OSA severity, and treatment adherence. Future prospective studies should combine systematic pre‐ablation sleep testing, objective CPAP adherence data, measures of nocturnal hypoxemia and hypoxic burden, standardized rhythm monitoring, and ablation‐modality stratification to define which OSAS phenotypes remain at increased risk after modern PVI.

## Funding

The authors have nothing to report.

## Conflicts of Interest

The authors declare no conflicts of interest.

## Data Availability

Data relating to this study are available upon reasonable request from the corresponding authors.
